# Oregano (*Origanum vulgare*) Extract Enhances Zebrafish (*Danio rerio*) Growth Performance, Serum and Mucus Innate Immune Responses and Resistance against *Aeromonas hydrophila* Challenge

**DOI:** 10.3390/ani11020299

**Published:** 2021-01-25

**Authors:** Ghasem Rashidian, Javad Tahmasebi Boldaji, Simona Rainis, Marko D. Prokić, Caterina Faggio

**Affiliations:** 1 Department of Aquaculture, Faculty of Marine Sciences, Tarbiat Modares University, Noor 4641776489, Iran; ghasemrashidiyan@gmail.com; 2 Dipartimento di Scienze Biomolecolare (DISB), Facoltà di Farmacia, Università degli Studi di Urbino “Carlo Bo”, Via Aurelio Saffi 2, 61029 Urbino, PU, Italy; javi.tahmasebi@yahoo.com; 3 Palmanova 62, 33028 Tolmezzo, UD, Italy; s.rainis@libero.it; 4 Department of Physiology, Institute for Biological Research “Siniša Stanković”, National Institute of Republic of Serbia, University of Belgrade, 11060 Belgrade, Serbia; marko.prokic@ibiss.bg.ac.rs; 5 Department of Chemical, Biological, Pharmaceutical and Environmental Sciences, University of Messina, Viale Ferdinando Stagno d’Alcontres 31, 98166 Messina, ME, Italy

**Keywords:** zebrafish, fish model, medicinal plant extracts, *Origanum vulgare*, ultrasound-assisted extraction, bacterial challenge, innate immunity

## Abstract

**Simple Summary:**

There has been extensive research addressing the positive effects of medicinal plant extracts using food fish as animal models. The present research was an attempt to assess zebrafish viability, considering the 3Rs approach, as an animal model for dietary experiments for fish nutrition studies, particularly evaluating novel additives for further implications in food fish. We have found oregano (*Origanum vulgare*) extract to be remarkably effective on fish growth and the fish immune system, enabling fish to fight a bacterial invasion.

**Abstract:**

This study evaluated the dietary effects of an ultrasound-assisted extract of *Origanum vulgare* on the growth, antioxidant and immune responses (serum and mucosal) and resistance of zebrafish (*Danio rerio*) against *Aeromonas hydrophila*. Four hundred and forty adult zebrafish were distributed into 12 tanks and fed 4 experimental diets including 0% (control), 0.5% (M1), 1% (M2) and 2% (M3) of the extract per kg^−1^ diet for eight weeks. Fish were then challenged with *A. hydrophila* and mortality was recorded for 10 days. Results revealed that the extract exerted potent effects on growth parameters of weight gain and specific growth rate. The feed conversion ratio was significantly lower in fish fed extract-incorporated diets. *O. vulgare* extract improved antioxidant and immune responses, resulting in less sensitivity to oxidative stress and a higher survival rate when challenged with *A. hydrophila*. Overall, the greatest effects were observed in individuals with 1% dietary inclusion of the extract. These results suggest that the extract from the plant *Origanum vulgare* possesses a great potential to be used in the aquaculture industry and that zebrafish is an appropriate model for nutrition studies.

## 1. Introduction

In fish, the primary lines of non-specific defenses are the skin and mucus supported by several soluble factors, such as activities of complements and lysozymes. The use of medicinal plants in aquaculture has been widely studied, implying their great potential to enhance growth, immunity and resistance against various experimental bacterial infections [1,2,3,4,5]. This suggests the great potential of natural compounds for substituting synthetic drugs and antibiotics in aquaculture. In particular, the remarkable role of medicinal plants has been recently discovered in aquaculture; in fact, they can stimulate the immune system, conferring early activation of non-specific defense mechanisms of fish. Medicinal plants archive their positive effects due to their contents such as polysaccharides, tannins, pigments, steroids, terpenoids, flavonoids, phenolic compounds, organic acids, alkaloids, glycosides and essential volatile oils [3,6,7,8,9]. Certain medicinal plants (herbs) possessing well-known immunostimulant effects have been used experimentally as well as clinically to treat various fish diseases and to control infections, for example, the septicemia caused by the bacteria *Aeromonas hydrophila*. 

*A. hydrophila* is widespread and economically relevant and this bacterium has been associated with disease in zebrafish (*Danio rerio*), salmonids (*Salmonidae*), carp (*Cyprinus carpio*), eels (*Anguilliformes* ssp.), milkfish, (*Chanos chanos*), channel catfish (*Ictalurus punctatus*), tilapia (*Oreochromis niloticus*) and ayu (*Plecoglossus altivelis*), and it can also be an opportunist in stress-related diseases [3]. In tilapia (*Oreochromis mossambicus*), effects on the non-specific humoral (lysozyme, antiprotease and complement) and cellular (myeloperoxidase content, production of reactive oxygen and nitrogen species) responses and disease resistance against *A. hydrophila* were evident. The relative percentage survival (RPS) values of tilapia, in the same clinical conditions, were enhanced by the administration of *Lonicera japonica* and *Ganoderma lucidum*, thanks to their positive effects on the non-specific immune response, particularly on the respiratory burst activity of white blood cells (WBC), phagocytosis, plasma lysozyme, total protein and total immunoglobulin contents [10]. Encouraging evidence was obtained also by the employment of Astragalo (*Astragalus membranaceus*) that enhanced lysozyme activities and skullcaps (*Scutellaria spp.*), which significantly inhibited extracellular superoxide anion production [11].

While developing vaccines is not often applicable and cost-effective [2], naturally occurring plant-based bioactive substances represent a very important tool to control diseases in aquaculture by enhancing the innate immunity of target animals. With respect to production costs, vaccines may often be expensive to produce for fish species that may be considered of low value in comparison to other species. Vaccines can protect fish from one or more pathogen and using adjuvants to boost the effectiveness would be required. Furthermore, intramuscular injection of vaccines is the most effective delivery method compared to oral and immersion methods that may not often work for fish of smaller sizes.

Nevertheless, in vivo experiments are inevitable to confirm the efficacy of potential benefits of novel feed additives and supplements, i.e., plant extracts, on the growth and immunity of fish; hence, using zebrafish as the model organism will drastically reduce the costs of such experiments. Zebrafish can be held in a small aquarium where it is more applicable to control and refine effective environmental factors. Nevertheless, small amounts of water will be used, and the zebrafish is not for human consumption. In addition, numerous experiments have been conducted on zebrafish (*Danio rerio*), as an animal model, employing various natural compounds with the aim of reducing antibiotic use and subsequently the risk of developing antibiotic-resistant strains of bacteria [12,13,14].

In the present experiment, we used the oregano (*Origanum vulgare*) plant from the family of *Lamiaceae*, commonly known as mountain mint in Iran and widely distributed throughout the Mediterranean area and Asia [15]. Oregano is reported to contain high levels of 0.15 to 1% essential oils with carvacrol as the main component (40 to 70% depending on the origin), flavonoids such as naringin and phenolic compounds [16]. It is often used both as a food flavor and in human medicine to treat gastric disorders, respiratory allergies, diabetes and healing wounds and as a sedative [17]. In addition to in vivo studies in rainbow trout (*Oncorhynchus mykiss)* [18] and gilthead seabream (*Sparus aurata*) [19], Oregano has been shown to exhibit antifungal, antioxidant and antibacterial activities in vitro [20,21], Authors have attributed these biological activities of the plant to the high content of phenolic compounds and volatile oils [16,18,19,20,21]. Even though essential oils from *O. vulgare* have been widely investigated in different species, few studies have addressed the effects of the plant extract on aquatic animals. To the best of our knowledge, there is no information available regarding the effects of *Origanum vulgare* plant extract obtained using an ultrasound-assisted extraction method on zebrafish growth and immunity. 

The objective of this study was to examine the effects of an ultrasound-assisted ethanolic extract of oregano on the growth performance, serum and mucus immune responses and resistance of zebrafish challenged with *A. hydrophila*.

## 2. Materials and Methods 

### 2.1. Plant Materials and Extraction

Fresh wild oregano plants were collected from Chalus (Mazandaran, Iran), air-dried in ambient temperature for 48 h and finely powdered using a SCH-800 grinder (Sunny, Japan). Ultrasonic-assisted extraction was performed according to Oroian et al. [22] with some modifications. In brief, 25 g of the dry powder was dissolved in 100 mL of 70% ethanol and sonicated at 30 kHz three times each for 15 min, and the resulting solution was thoroughly mixed with a stirrer (50 °C and 150 rpm) to avoid sedimentation and allow proper extraction in dark conditions for 24 h. The obtained solution was filtered through a Whatman no. 1 filter, concentrated using a rotary evaporator and freeze-dried (D-37520 Osterode, Germany), being kept at 4 °C until further investigations. Total phenolic compounds and total flavonoids of the extract were measured according to Mcdonald [23] and Zhishen [24] as 153 ± 11.08 and 81.46 ± 6.11 mg g^−1^ extract, respectively.

### 2.2. Diets Preparation and Feeding Protocol

Feed ingredients were provided from commercial sources and diets were formulated to contain desired levels of 0, 0.5, 1 and 2% of the extract per kg^−1^ diet added to the smooth paste at the final stage, respectively, named as Control, M1, M2 and M3 (Table 1). The powdered extract was dissolved in distilled water along with gelatin and mixed up with the other diet components. Diets were cooled at −20 °C, pelleted using an industrial grinder and sieved with 1.2 mm mesh size. Diets were then packed and stored at 4 °C until use. Fish were fed 3% of their respective body weight three times a day for eight weeks. On the third and sixth weeks, fish were bulk weighed and the feeding rate was adjusted accordingly.

### 2.3. Fish Supply and Rearing Conditions

The experiment was carried out through a completely randomized design including four treatments in triplicate in accordance with the National Institutes of Health guide for the care and use of laboratory animals (NIH Publications No. 8023, revised 1978). All animal procedures complied with the EC Directive and 86/609/EEC European Directive (2010/63/EU) on the protection of animals used for experimental and other scientific purposes. A total 240 adult zebrafish (male and female) with no history of health problems were obtained from a local farm in Karaj, Iran. Fish were allowed to adapt to laboratory conditions for 1 week and were fed on a basal diet (control diet). Water was filtered automatically and monitored daily. Water quality was adjusted if necessary and physicochemical parameters of hardness (KH), temperature, pH, dissolved oxygen, NO_2_, NO_3_ and NH_4_ were measured as 79 ± 8.35 ppm, 26 ± 1 °C, 6.8 ± 0.46, 7.42 ± 0.37 ppm, 5.3 ± 1.14 ppm, 13.5 ± 4.22 ppm, and 0.62 ± 0.08 ppm, respectively.

### 2.4. Growth Performance

At the end of the trial, weight gain (WG), food conversion ratio (FCR), specific growth rate (SGR) and survival rate (SR) of each experimental unit were calculated according to the formulas below:

WG (g) = Final weight − Initial weight,(1)

FCR = Feed intake/Weight gain,(2)

SGR (% day^−1^) = [(log_n_ final weight − log_n_ initial weight)/experimental period (days)] × 100, (3)

SR (%) = [(final number of fish)/(initial number of fish)] ×100,(4)

Fish were bulk weighed and the mean of three measurements was considered for initial and final weight values [25,26].

### 2.5. Serum and Skin Mucus Immunity

#### 2.5.1. Sample Collection

After 24-h starvation on 55th day of the experiment, 10 fish were randomly taken from each replicate, anesthetized with iced water using ice chips and prepared for serum collection, according to the protocol described by Pedroso et al. [27]. The remaining specimens were transferred to new tanks for the infection experiment. Samples were pooled in sterile new microtubes and centrifuged for 5 min at 5000 g and 4 °C. The supernatant was carefully separated and kept at −20 °C for further analysis. Mucus was collected by gently rubbing the anesthetized fish skin using a glass slide and mucus samples were diluted in phosphate buffer (pH = 7, Merck). The samples were centrifuged at 1500 g for 5 min and the supernatant was transferred to −80 °C for further evaluation.

#### 2.5.2. Lysozyme Activity

Serum and mucus lysozyme activity was measured according to Ellis [28] with slight adjustments. For calibration, a standard curve was provided using chicken egg white lysozyme (Sigma-Aldrich Corp. - St Louis, MO, USA) following a turbidimetric method, where serum aliquots in a 0.05 M sodium phosphate buffer (pH 6.2) were added to a *Micrococcus luteus* (0.6 mg mL^−1^) suspension. All reactions were run at room temperature and absorbance was read spectrophotometrically at 450 nm every 3 min for 15 min.

#### 2.5.3. Total Immunoglobulin (Total Ig) 

The total Ig content of serum samples was estimated following the modified method described by Milla [29], which involves subtracting the total protein content of serum before and after protein precipitation with 12% polyethylene glycol (PEG, 10,000 MW, Sigma-Aldrich Corp. - St Louis, MO, USA).

#### 2.5.4. Complement Activity

Alternative complement activity (ACH50) was determined based on the hemolysis of sheep red blood cells (SRBC) according to Yano [30]. The volume of serum yielding 50% hemolysis was determined and used to calculate the complement activity of the samples (value of ACH50 is in unit mL^−1^).

#### 2.5.5. Enzymes Activity

The activity of alkaline phosphatase (ALP, Pars Azmun Co., Tehran, Iran), superoxide dismutase (SOD, NS-15032, Nasdox), catalase (CAT, NS-15052, Nasdox, Tehran, Iran) and protease was measured using commercial kits (Pars Azmun, Iran) following the manufacturer’s instructions.

#### 2.5.6. Total Protein

Total protein (TP) content was quantified using available commercial kits (Pars Azmun, Iran) following the manufacturer’s instructions.

#### 2.5.7. Malondialdehyde (MDA) Content

MDA content was determined using a thiobarbituric acid (TBA) method according to Lovrić [31] with slight modifications. In this method, MDA level was measured spectrophotometrically at 540 nm during the thiobarbituric acid reaction and values are reported as nmol mg^−1^.

### 2.6. Bacterial Challenge

Five identical colonies of *A. hydrophila* (RTCC1032) were cultured in broth medium and bulked to achieve maximum concentration in 36 h. For the challenge experiment, after 8 weeks of the dietary experiment, 20 fish from each tank were transferred to new tanks and fed on a basal diet that was 3% of their body weight, water was reduced to half and fish were immersed in 1.7 × 10^6^ CFU mL^−1^ concentration of the bacterium. The concentration was chosen according to previous infection studies on zebrafish and our primary lab experiences where LC50_24h_ of *A. hydrophila* was measured around 0.6 to 0.8 × 10^7^ CFU mL^−1^. Challenge tanks were provided with air stones in order to supply oxygen and keep the bacteria suspended for maximum interaction. After 24-h exposure, water was entirely exchanged twice, and dead fish were removed immediately if there were any for a period of 10 days.

### 2.7. Statistical Analysis

Possible outliers were checked by Grubb’s test. Assumptions of normality (Kolmogorov–Smirnov test) and homogeneity of variances (Levine’s test) were respected. Possible differences between control and experimental diets were analyzed using one-way ANOVA. The post hoc LSD was performed to determine further differences for each variable. The significance level was set as *p* < 0.05. All statistical analyses were performed using STATISTICA version 8.0. (StatSoft, Inc., Tulsa, OK, USA).

## 3. Results

### 3.1. Growth Performance

Measured growth parameters are shown in Table 2. Initial fish weights did not differ significantly. After eight weeks, 1% *O. vulgare* extract (M2-group) statistically improved fish final weight and SGR in comparison to all other groups, while FCR values were improved only in comparison to the control diet. Weight gain was statistically enhanced in individuals belonging to M2 and M3 groups. No mortality was recorded during the eight weeks of the experiment in all treatments. Overall, the best results of growth performance were obtained in the M2 treatment.

### 3.2. Immunological and Antioxidant Response Parameters

**Figure 1:**
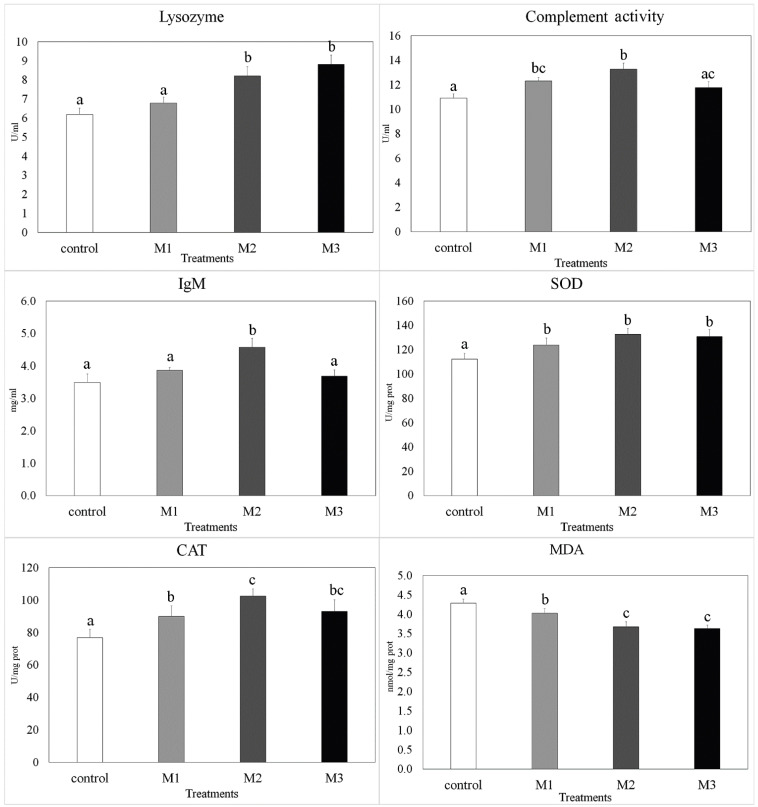
Serum immunological response (lysozyme, complement activity, total Ig), antioxidant enzymes activity (SOD—superoxide dismutase, CAT—catalase) and MDA (malondialdehyde) levels of zebrafish (*Danio rerio*) fed on four experimental diets: 0% (control), 0.5% (M1), 1% (M2) and 2% (M3) inclusion of the extract per kg diet for eight weeks. Different letters indicate significant differences between treatments (*p* < 0.05). Data represent mean ± SE.

Serum immunological and biochemical parameters are presented in Figure 1. Lysozyme activity was statistically higher in M2 and M3 group individuals as compared to the other two. The total immunoglobulin value was only significantly increased in the M2 group. As for the complement, we observed a higher value in individuals of the M2 group than control and M3 group individuals. Values for complement activity in fish belonging to the M1 group were also higher than the control. Total protein in the M2 (3.43 ± 0.39 g/dl) and M3 (3.17 ± 0.19 g/dl) groups was higher than in the control group (2.46 ± 0.23 g/dl) (*p* = 0.0021 and *p* = 0.011, respectively). M2 individuals also had higher values for total protein in comparison to M1 group (2.81 ± 0.20 g/dl) fishes (*p* = 0.021). Antioxidant enzyme SOD showed higher activity in all treated groups in comparison to the control. CAT activity was highest in the M2 group followed by the M3 and M1 groups and lowest in the control individuals. MDA, as a biomarker of lipid peroxidation, had the highest concentrations in the control group. A comparison between treatments showed that M1 had a higher concentration than the other two groups.

### 3.3. Mucus Immune Responses

Mucosal immune parameters such as lysozyme, ALP, total Ig, protease activity and total protein are presented in Table 3. Results showed that ALP and lysozyme activity of mucus were remarkably affected by experimental treatments in comparison to the control. A comparison between treatments revealed that group M2 had significantly higher values for both parameters than M1 and M3. Protease activity was increased in M2 individuals as compared to the control and M3. For total Ig, all examined groups differed, where the highest was in M2, followed by M3, then M1, and the lowest was for the control group. Individuals from the M2 group also displayed the highest concentrations of total protein. Higher concentrations were also reported for the M3 group when compared to the control.

### 3.4. Survival Rate

**Figure 2:**
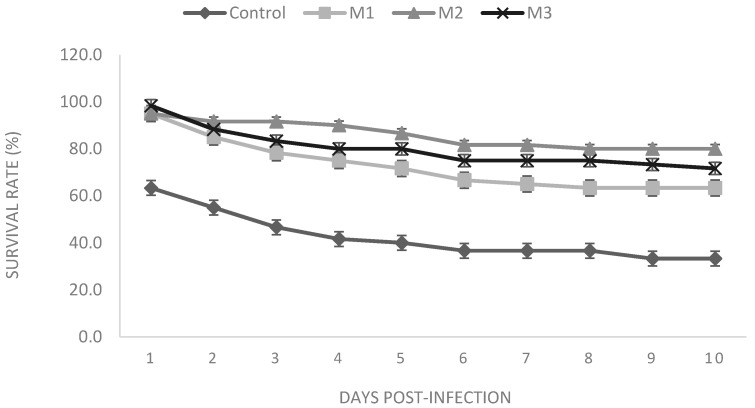
The survival rate of zebrafish (*Danio rerio*) challenged with A. hydrophila after 10 days. Fish were fed on four experimental diets: 0% (control), 0.5% (M1), 1% (M2) and 2% (M3) inclusion of the extract per kg^−1^ diet for eight weeks.

Results from the challenge experiment are shown in Figure 2. We observed that fish receiving different inclusion levels of *O. vulgare* extract could resist the infection more than the control group, with the M2 treatment showing the highest survival rate.

## 4. Discussion

There has been a growing interest in zebrafish (*Danio rerio*) as a model animal in different disciplines [32] and it has been suggested as an appropriate model organism to evaluate the potential of feed additives in aquaculture [33,34]. Thus, the present experiment was aimed to investigate dietary effects of different levels of 0% (control), 0.5% (M1), 1% (M2) and 2% (M3) kg^−1^ of medicinal plant *O. vulgare* on zebrafish (*D. rerio*) growth and immunity. Our findings confirm the basic idea and further develop the idea of the initial screening of *O. vulgare* plant extracts for aquaculture purposes using zebrafish. Regardless of present bioactive substances, the plant has been reported to have in vitro antioxidant activities [16,35,36,37] and in vivo antioxidant and immunomodulatory effects have been confirmed in further studies [18,38,39,40].

In this trial, an ultrasound-assisted extraction method using ethanol as the solvent was employed to provide an extract with a high content of antioxidant flavonoids (81.46 ± 6.11 mg g^−1^) and polyphenols (153 ± 11.08 mg g^−1^) from the medicinal plant *O. vulgare*. Ultrasound-assisted extraction was employed because it is suggested to improve solvent extraction method issues such as long duration, toxic solvents, environmental contamination and low yield output [41]. It is worth mentioning that the plant’s proximate composition may differ from one origin to another, reflecting on their biological activities. 

Although numerous studies have demonstrated the positive effects of plant extracts on the growth performance of different fish species, the underlying mechanism is not fully understood. Eight weeks of the dietary trial of *O. vulgare* extract enhanced zebrafish (*D. rerio*) growth and innate immune responses with better values found in fish fed moderate inclusion levels of 1% of the extract. This might be due to activations or deactivations at molecular levels unknown to us since there are numerous substances scarcely present in the plant extract that we did not trace. In fact, M2-1% treatment showed growth parameters of weight gain, FCR and SGR to be significantly improved; however, other treated groups presented rather mild alterations. In contrast to the present results, Ahmadifar et al. [42] did not find any remarkable improvement in zebrafish (*D. rerio*) growth parameters fed dietary ginger extract for eight weeks. Nonetheless, plant extracts are utilized in aquaculture because they are cost-effective and there are no reports regarding hazardous environmental impacts [43,44].

Harikrishnan and colleagues [45] suggested that a higher dosage of medicinal plants in the diet may suppress the immune system while enhancing disease resistance. We observed no mortality during the dietary trial and improved immunological and antioxidant responses in moderate levels of incorporation of the extract (1%). The effective dose depends on the plant, fish species and administration method. In this experiment, the extract was administered through the diet, which is suggested as the most practical method of exploiting medicinal plants in aquaculture [11,45], and we found the 1% inclusion level to be the most effective on the measured immune parameters. Some authors found similar results with 1% dietary cinnamon, and 1.25% of caraway seed meals were outlined as the most effective doses on Nile tilapia (*Tilapia niloticus*) [46,47]. In another experiment conducted on zebrafish, Ahmadifar and colleagues [42] reported that 2 and 3% of ginger extract were more effective on immune parameters of lysozyme, complement activity and total immunoglobulin.

Lysozyme is an important protein produced particularly by macrophages and neutrophils which is able to lyse Gram+ and Gram^−^ bacteria and increase phagocytosis [48]. In addition, this protein is responsible for activating other important molecules of defense, including the complement system and phagocytic cells [28]. The level of lysozyme activity depends on environmental parameters (water temperature, pH, light period, season and toxins) and intrinsic factors (size, age, sex, infections and stress) [49]. Our findings show that serum lysozyme activity was significantly increased in fish fed with 1 and 2% diets of oregano. Similar results have been reported using oregano (*O. vulgare*) in rainbow trout (*Oncorhynchus mykiss*) [18] and other plant extracts in different species [34,42,50,51,52,53]. Ahmadifar et al. [54] attributed the increased lysozyme activity to the upregulation of the lysozyme-encoding gene. ALP activity, protease and total Ig were also affected by the extract and the M2 group was more effective. There are controversial reports regarding the effects of plant extracts on serum total protein [53]. However, the present results show an increase in total protein levels of fish fed oregano extract.

The MDA level is a biological marker of lipid peroxidation in which, under oxidative stress, the MDA level is drastically increased. In the present experiment, decreased MDA levels of fish fed dietary oregano extract can be linked with boosted first-line enzymes of the antioxidative defense (SOD and CAT) involved in the prevention of reactive oxygen species formation (SOD superoxide radical and CAT-H_2_O_2_). The antioxidant nature of the extract seen as a reduction in MDA levels in the treatment groups compared to the control was also related to an increase in the level of antioxidant enzyme activities [16,55]. Similar results were found in rainbow trout (*Oncorhynchus mykiss*) fed oregano (*O. vulgare*) extract [55] and zebrafish fed quercetin [56]. Lower oxidative stress, beside direct positive effects on a fish organism’s health, can also positively affect fitness parameters of growth, development, longevity and reproduction.

Alteration in serum total protein can be used in evaluating the health status of aquatic animals [57,58]. In the present experiment, total protein was significantly increased in fish fed oregano extract, which may be due to the enhancement of innate immune responses.

After eight weeks of the dietary trial, fish receiving *O. vulgare* in their diets showed an improved survival rate when compared to that of the control treatment after 10 days post-infection with *A. hydrophila*. To support the possible effects of oregano extract on enhanced immune defense against bacterial infection, Teixeira et al. [16] found the ethanolic extract of the plant effective on a number of bacteria. In addition, based on present results, zebrafish immunity was merely enhanced, and heightening fish innate immune defense enabled them to resist challenge tests. Similar results have been reported using different extracts in different fish species [59]. 

Several studies have reported dietary plant extracts to be effective on fish immunological responses due to their respective bioactive components [18,55,60,61,62]. Although further research is required to identify mechanisms of actions of exerted growth and immune enhancement effects of oregano extract, authors suggest high levels of polyphenols and flavonoids in *O. vulgare* extract are possibly responsible for the observed positive effects. Since in vivo experiments are inevitable to confirm the efficacy of potential benefits of novel feed additives and supplements on the growth and immunity of fish, zebrafish is recommended as a model organism for further discovery of the potential of plant extracts for aquaculture purposes. Using zebrafish as a model organism will drastically reduce the costs of such experiments. Zebrafish can be held in a small aquarium where it is more applicable to control and refine effective environmental factors. Nevertheless, small amounts of water will be used, and the zebrafish is not for human consumption.

## 5. Conclusions

In conclusion, our results reveal that *O. vulgare* could lower oxidative stress and enhance serum and mucus immune responses in treated fish. It is interesting to underline that a medium concentration of *O. vulgare* guarantees the same results of the higher one bearing the best productive performance in the meantime. The present findings in zebrafish (*D. rerio*) are aligned with those found in other food fish. Our findings suggest *O. vulgare* as a dietary mean to enhance fish resistance, particularly against the tested bacterium of *A. hydrophila*. Therefore, considering the ease of handling and experiment expenses, the authors would like to suggest zebrafish (*D. rerio*) as an animal model for the further initial discovery of plant extracts’ potential and other bioactive substances in aquaculture.

## Figures and Tables

**Table 1:** Proximate composition of experimental diets including different levels of *O. vulgare* extract.

Proximate Composition (%)	Control	M1 (0.5%)	M2 (1%)	M3 (2%)
Dry matter	92.44	92.14	92.48	92.66
Ash	13.66	12.99	13.42	13.84
Crude protein	51.69	50.97	51.48	52.19
Crude lipids	11.94	11.92	11.53	11.25

**Table 2:** Growth parameters (WG: weight gain; SGR: specific growth rate; FCR: feed conversion ratio; SR: survival rate) of zebrafish (*Danio rerio*) fed on four experimental diet regimes: 0% (control), 0.5% (M1), 1% (M2) and 2% (M3) inclusion of the extract per kg diet for eight weeks.

Growth Parameters	Control	M1 (0.5%)	M2 (1%)	M3 (2%)
Initial weight (mg)	81.13 ± 0.87 ^a^	82.58 ± 1.17 ^a^	81.62 ± 1.05 ^a^	81.03 ± 1.27 ^a^
Final weight (mg)	190.46 ± 1.87 ^a^	194.52 ± 1.97 ^a^	199.19 ± 2.9 ^b^	194.33 ± 1.77 ^a^
WG (mg)	109.32 ± 1.15 ^a^	111.93 ± 2.23 ^a^	117.57 ± 3.94 ^b^	113.30 ± 1.07 ^ab^
WG (%)	134.74 ± 1.12 ^a^	135.57 ± 4.00 ^a^	144.10 ± 6.67 ^b^	139.83 ± 2.41 ^ab^
FCR	1.92 ± 0.06 ^a^	1.87 ± 0.03 ^ab^	1.82 ± 0.03 ^b^	1.85 ± 0.02 ^ab^
SGR (%/d)	1.42 ± 0.01 ^a^	1.43 ± 0.03 ^a^	1.49 ± 0.05 ^b^	1.46 ± 0.02 ^ab^
SR (%)	100 ± 0	100 ± 0	100 ± 0	100 ± 0

Different letters indicate significant differences between treatments (*p* < 0.05). Data represent mean ± SE.

**Table 3:** Mucosal immune parameters: lysozyme, ALP, total Ig, protease activity and total protein in zebrafish fed on four experimental diet regimes: 0% (control), 0.5% (M1), 1% (M2) and 2% (M3) inclusion of the extract per kg diet for eight weeks.

Treatment	Control	M1 (0.5%)	M2 (1%)	M3 (2%)
Lysozyme (U mL^−1^)	31.23 ± 0.67 ^a^	35.71 ± 1.34 ^b^	41.20 ± 1.93 ^c^	35.11 ± 1.45 ^b^
ALP (U mL^−1^)	12.83 ± 1.68 ^a^	17.92 ± 1.54 ^b^	23.66 ± 2.23 ^c^	20.37 ± 0.87 ^b^
Protease	24.88 ± 1.77 ^a^	27.88 ± 1.19 ^ab^	31.04 ± 2.06 ^b^	25.79 ± 1.63 ^a^
Total Ig (mg mL^−1^)	9.28 ± 0.85 ^a^	12.13 ± 0.80 ^b^	16.81 ± 1.03 ^c^	15.06 ± 0.72 ^d^
Total protein (mg mL^−1^)	4.26 ± 0.33 ^a^	4.61 ± 0.30 ^ab^	5.70 ± 0.35 ^c^	5.10 ± 0.24 ^b^

Different letters indicate significant differences between treatments (*p* < 0.05). Data represent mean ± SE.

## Data Availability

Data available on request due to restrictions, e.g., privacy or ethical. The data presented in this study are available on request from the corresponding author. The data are not publicly available due to the law of the Ministry of Science Research and Technology.

## References

[1] Immanuel G., Vincybai V.C., Sivaram V., Palavesam A., Marian M. (2004). Effect of butanolic extracts from terrestrial herbs and seaweeds on the survival, growth and pathogen (*Vibrio parahaemolyticus*) load on shrimp *Penaeus indicus* juveniles. Aquaculture.

[2] Pandey G., Sharma M. (2012). Immunostimulant effect of medicinal plants on fish. Int. Res. J. Pharm..

[3] Reverter M., Tapissier-Bontemps N., Sarter S., Sasal P., Caruso D. (2020). Moving towards more sustainable aquaculture practices: A meta-analysis on the potential of plant-enriched diets to improve fish growth, immunity and disease resistance. Rev. Aquac..

[4] Van Hai N. (2015). The use of medicinal plants as immunostimulants in aquaculture: A review. Aquaculture.

[5] Elumalai P., Kurian A., Lakshmi S., Faggio C., Esteban M.A., Ringø E. (2020). Herbal Immunomodulators in Aquaculture. Rev. Fish. Sci. Aquac..

[6] Raman P.R. (2017). Applicability, feasibility and efficacy of phytotherapy in aquatic animal health management. Am. J. Plant Sci..

[7] Stratev D., Zhylyazkov G.I., Noundu X.S., Krause R. (2008). Beneficial effects of medicinal plants in fish diseases. Aquac. Int..

[8] Harikrishnan R., Moon Y.-G., Kim M.-C., Kim J.-S., Heo M.-S., Balasundaram C., Dharaneedharan S. (2010). Phytotherapy of ae romonas hydrophila-infected goldfish, carassius auratus. J. World Aquac. Soc..

[9] Chakraborty S.B., Hancz C. (2011). Application of phytochemicals as immunostimulant, antipathogenic and antistress agents in finfish culture. Rev. Aquac..

[10] Wang C., Liu H., Mu G., Lu S., Wang D., Jiang H., Sun X., Han S., Liu Y. (2019). Effects of traditional Chinese medicines on immunity and culturable gut microflora to *Oncorhynchus masou*. Fish Shellfish. Immunol..

[11] Doan H.V., Soltani E., Ingelbrecht J., Soltani M. (2020). Medicinal herbs and plants: Potential treatment of monogenean infections in fish. Rev. Fish. Sci. Aquac..

[12] Ardó L., Guojun Y., Jeney Z., Pao X., Jeney G. (2007). Effect of fish feed containing two Chinese herbal extracts (Ganodema Iucidum and *Lonicera japonica*) on the non-specific immune system of *Nile tilapia*, *Oreochromis niloticus* (preliminary results). Acta Agrar. Debr..

[13] Yin G., Jeney G., Racz T., Xu P., Jun X., Jeney Z. (2006). Effect of two Chinese herbs (*Astragalus radix* and *Scutellaria radix*) on non-specific immune response of tilapia, *Oreochromis niloticus*. Aquaculture.

[14] Plhalova L., Sehonova P., Blahová J., Doubkova V., Tichy F., Faggio C., Berankova P., Svobodova Z. (2020). Evaluation of tramadol hydrochloride toxicity to juvenile zebrafish—morphological, antioxidant and histological responses. Appl. Sci..

[15] Blahova J., Cocilovo C., Plhalova L., Svobodova Z., Faggio C. (2020). Embryotoxicity of atrazine and its degradation products to early life stages of zebrafish (*Danio rerio*). Environ. Toxicol. Pharmacol..

[16] Petrovici A., Strungaru S.-A., Nicoara M., Robea M.A., Solcan C., Faggio C. (2020). Toxicity of deltamethrin to zebrafish gonads revealed by cellular biomarkers. J. Mar. Sci. Eng..

[17] Vokou D., Kokkini S., Bessiere J.-M. (1993). Geographic variation of Greek oregano (*Origanum vulgare* ssp. *hirtum*) essential oils. Biochem. Syst. Ecol..

[18] Teixeira B., Marques A., Ramos C., Serrano C., Matos O., Neng N.R., Nogueira J.M.F., Saraiva J.A., Nunes M.L. (2013). Chemical composition and bioactivity of different oregano (*Origanum vulgare*) extracts and essential oil. J. Sci. Food Agric..

[19] Morshedloo M.R., Pirali Hamedani M., Yazdani D. (2018). An over review to *Origanum vulgare* L. and its pharmacological properties. J. Med. Plant..

[20] Bulfon C., Volpatti D., Galeotti M. (2014). In vitro antibacterial activity of plant ethanolic extracts against fish pathogens. J. World Aquac. Soc..

[21] Beltrán J.M.G., Espinosa C., Guardiola F.A., Esteban M.Á. (2018). In vitro effects of *Origanum vulgare* leaf extracts on gilthead seabream (*Sparus aurata* L.) leucocytes, cytotoxic, bactericidal and antioxidant activities. Fish Shellfish. Immunol..

[22] Pourmoghim H., Haghighi M., dan Rohani M.S. (2015). Effect of dietary inclusion of *Origanum vulgare* extract on nonspecific immune response and hematological parameters of rainbow trout (*Oncorhynchusmykiss*). Bull. Environ. Pharmacol. Life Sci..

[23] Beltrán J.M.G., Silvera D.G., Ruiz C.E., Campo V., Chupani L., Faggio C., Esteban M.Á. (2020). Effects of dietary *Origanum vulgare* on gilthead seabream (*Sparus aurata* L.) immune and antioxidant status. Fish Shellfish. Immunol..

[24] Oroian M., Dranca F., Ursachi F. (2020). Comparative evaluation of maceration, microwave and ultrasonic-assisted extraction of phenolic compounds from propolis. J. Food Sci. Technol..

[25] McDonald S., Prenzler P.D., Antolovich M., Robards K. (2001). Phenolic content and antioxidant activity of olive extracts. Food Chem..

[26] Zhishen J., Mengcheng T., Jianming W. (1999). The determination of flavonoid contents in mulberry and their scavenging effects on superoxide radicals. Food Chem..

[27] Du R.-Y., Chen J.-X., Zhu J., Feng J.-Y., Luo L., Lin S.-M., Chen Y.-J. (2020). Glucose homeostasis and glucose tolerance were impaired with elevated lipid to starch ratios in practical diets for the omnivorous genetically improved farmed tilapia *Oreochromis niloticus*. Aquaculture.

[28] Sun S., Ye J., Chen J., Wang Y., Chen L. (2010). Effect of dietary fish oil replacement by rapeseed oil on the growth, fatty acid composition and serum non-specific immunity response of fingerling black carp, *Mylopharyngodon piceus*. Aquac. Nutr..

[29] Pedroso G.L., Hammes T.O., Escobar T.D., Fracasso L.B., Forgiarini L.F., da Silveira T.R. (2012). Blood collection for biochemical analysis in adult zebrafish. J. Vis. Exp..

[30] Ellis A.E., Stolen J.S., Fletcher T.C., Anderson D.P., Roberson B.S., van Muiswinkel W.B. (1990). Lysozyme assays. Techniques in Fish Immunology.

[31] Yano T., Iwama G., Nakahishi T. (1996). The Nonspecific Immune System: Humoral Defense. Fish Physiology.

[32] Lovrić J., Mesić M., Macan M., Koprivanac M., Kelava M., Bradamante V. (2008). Measurement of malondialdehyde (MDA) level in rat plasma after simvastatin treatment using two different analytical methods. Period. Biol..

[33] Strähle U., Scholz S., Geisler R., Greiner P., Hollert H., Rastegar S., Schumacher A., Selderslaghs I., Weiss C., Witters H. (2012). Zebrafish embryos as an alternative to animal experiments—A commentary on the definition of the onset of protected life stages in animal welfare regulations. Reprod. Toxicol..

[34] Sullivan C., Kim C.H. (2008). Zebrafish as a model for infectious disease and immune function. Fish Shellfish. Immunol..

[35] Safari R., Hoseinifar S.H., Van Doan H., Dadar M. (2017). The effects of dietary Myrtle (*Myrtus communis*) on skin mucus immune parameters and mRNA levels of growth, antioxidant and immune related genes in zebrafish (*Danio rerio*). Fish Shellfish. Immunol..

[36] Yanishlieva N.V., Marinova E.M., Gordon M.H., Raneva V.G. (1999). Antioxidant activity and mechanism of action of thymol and carvacrol in two lipid systems. Food Chem..

[37] Lagouri V., Boskou D. (1996). Nutrient antioxidants in oregano. Int. J. Food Sci. Nutr..

[38] Kulisic T., Radonic A., Katalinic V., Milos M. (2004). Use of different methods for testing antioxidative activity of oregano essential oil. Food Chem..

[39] Zheng Z., Tan J.Y.W., Liu H., Zhou X.H., Xiang X., Wang K.Y. (2009). Evaluation of oregano essential oil (*Origanum heracleoticum* L.) on growth, antioxidant effect and resistance against *Aeromonas hydrophila* in channel catfish (*Ictalurus punctatus*). Aquaculture.

[40] Abdel-Latif H.M.R., Khalil R.H. (2014). Evaluation of two Phytobiotics, *Spirulina platensis* and *Origanum vulgare* extract on Growth, Serum antioxidant activities and Resistance of Nile tilapia (*Oreochromis niloticus*) to pathogenic *Vibrio alginolyticus*. Int. J. Fish. Aquat. Stud..

[41] Diler O., Gormez O., Diler I., Metin S. (2017). Effect of oregano (*Origanum onites* L.) essential oil on growth, lysozyme and antioxidant activity and resistance against *Lactococcus garvieaein* rainbow trout, *Oncorhynchus mykiss* (Walbaum). Aquac. Nutr..

[42] Ahmadifar E., Sheikhzadeh N., Roshanaei K., Dargahi N., Faggio C. (2019). Can dietary ginger (*Zingiber officinale*) alter biochemical and immunological parameters and gene expression related to growth, immunity and antioxidant system in zebrafish (*Danio rerio*)?. Aquaculture.

[43] Jian J., Wu Z. (2003). Effects of traditional Chinese medicine on nonspecific immunity and disease resistance of large yellow croaker, *Pseudosciaena crocea* (Richardson). Aquaculture.

[44] Citarasu T. (2010). Herbal biomedicines: A new opportunity for aquaculture industry. Aquac. Int..

[45] Harikrishnan R., Balasundaram C., Heo M.-S. (2011). Impact of plant products on innate and adaptive immune system of cultured finfish and shellfish. Aquaculture.

[46] Ahmad M.H., El Mesallamy A.M.D., Samir F., Zahran F. (2011). Effect of Cinnamon (*Cinnamomum zeylanicum*) on growth performance, feed utilization, whole-body composition, and resistance to *Aeromonas hydrophilain* Nile Tilapia. J. Appl. Aquac..

[47] Ahmad M.H., Abdel-Tawwab M. (2011). The use of caraway seed meal as a feed additive in fish diets: Growth performance, feed utilization, and whole-body composition of Nile tilapia, *Oreochromis niloticus* (L.) fingerlings. Aquaculture.

[48] Alexander J.B., Ingram G.A. (1992). Noncellular nonspecific defence mechanisms of fish. Annu. Rev. Fish Dis..

[49] Tukmechi A., Bandboni M. (2014). Effects of Saccharomyces cerevisiae supplementation on immune response, hematological parameters, body composition and disease resistance in rainbow trout, *Oncorhynchus mykiss* (Walbaum, 1792). J. Appl. Ichthyol..

[50] Hoseinifar S.H., Yousefi S., Capillo G., Paknejad H., Khalili M., Tabarraei A., Van Doan H., Spanò N., Faggio C. (2018). Mucosal immune parameters, immune and antioxidant defence related genes expression and growth performance of zebrafish (*Danio rerio*) fed on *Gracilaria gracilis* powder. Fish Shellfish. Immunol..

[51] Farsani M.N., Hoseinifar S.H., Rashidian G., Ghafarifarsani H., Ashouri G., Van Doan H. (2019). Dietary effects of *Coriandrum sativum* extract on growth performance, physiological and innate immune responses and resistance of rainbow trout (*Oncorhynchus mykiss*) against *Yersinia ruckeri*. Fish Shellfish. Immunol..

[52] Rashidian G., Kajbaf K., Prokić M.D., Faggio C. (2020). Extract of common mallow (*Malvae sylvestris*) enhances growth, immunity, and resistance of rainbow trout (*Oncorhynchus mykiss*) fingerlings against *Yersinia ruckeri* infection. Fish Shellfish. Immunol..

[53] Dügenci S.K., Arda N., Candan A. (2003). Some medicinal plants as immunostimulant for fish. J. Ethnopharmacol..

[54] Ahmadifar E., Dawood M.A.O., Moghadam M.S., Sheikhzadeh N., Hoseinifar S.H., Musthafa M.S. (2019). Modulation of immune parameters and antioxidant defense in zebrafish (*Danio rerio*) using dietary apple cider vinegar. Aquaculture.

[55] Rafieepour A., Hajirezaee S., Rahimi R. (2020). Dietary oregano extract (*Origanum vulgare* L.) enhances the antioxidant defence in rainbow trout, *Oncorhynchus mykiss* against toxicity induced by organophosphorus pesticide, diazinon. Toxin Rev..

[56] Wang J., Zhang C., Zhang J., Xie J., Yang L., Xing Y., Li Z. (2020). The effects of quercetin on immunity, antioxidant indices, and disease resistance in zebrafish (*Danio rerio*). Fish Physiol. Biochem..

[57] Riche M. (2007). Analysis of refractometry for determining total plasma protein in hybrid striped bass (*Morone chrysops* × *M. saxatilis*) at various salinities. Aquaculture.

[58] Fazio F., Marafioti S., Torre A., Sanfilippo M., Panzera M., Faggio C. (2013). Haematological and serum protein profiles of *Mugil cephalus*: Effect of two different habitats. Ichthyol. Res..

[59] Suwan C., Noimoon P., Yawichai P., Jitmanowan S., Chitmanat C. (2020). Effects of medicinal plants on fish immunity and its growth performances. Burapha Sci. J..

[60] Mohammadi G., Rashidian G., Hoseinifar S.H., Naserabad S.S., Van Doan H. (2020). Ginger (*Zingiber officinale*) extract affects growth performance, body composition, haematology, serum and mucosal immune parameters in common carp (*Cyprinus carpio*). Fish Shellfish. Immunol..

[61] Aragona M., Lauriano E.R., Pergolizzi S., Faggio C. (2017). *Opuntia ficus-indica* (L.) Miller as a source of bioactivity compounds for health and nutrition. Nat. Prod. Res..

[62] Van Doan H., Hoseinifar S.H., Sringarm K., Jaturasitha S., Yuangsoi B., Dawood M.A.O., Esteban M.A., Ringø E., Faggio C. (2019). Effects of assam tea extract on growth, skin mucus, serum immunity and disease resistance of Nile tilapia (*Oreochromis niloticus*) against *Streptococcus agalactiae*. Fish Shellfish. Immunol..

